# One-month outcomes of patients with SARS-CoV-2 infection and their relationships with lung ultrasound signs

**DOI:** 10.1186/s13089-021-00223-9

**Published:** 2021-04-09

**Authors:** Thiago Thomaz Mafort, Rogério Rufino, Claudia Henrique da Costa, Mariana Soares da Cal, Laura Braga Monnerat, Patrícia Frascari Litrento, Laura Lizeth Zuluaga Parra, Arthur de Sá Earp de Souza Marinho, Agnaldo José Lopes

**Affiliations:** 1 Department of Pulmonology, Piquet Carneiro Policlinic, State University of Rio de Janeiro, Av. Mal. Rondon, 381, São Francisco Xavier, Rio de Janeiro, 20950-003 Brazil; 2 Postgraduate Programme in Medical Sciences, School of Medical Sciences, State University of Rio de Janeiro, Av. Prof. Manuel de Abreu, 444, 2° andar, Vila Isabel, Rio de Janeiro, 20550-170 Brazil; 3 Rehabilitation Sciences Post-Graduation Programme, Augusto Motta University Centre (UNISUAM), Rua Dona Isabel, 94, Bonsucesso, Rio de Janeiro, 21032-060 Brazil

**Keywords:** COVID-19, SARS-CoV-2, Lung ultrasound, Pneumonia, 1-month outcomes

## Abstract

**Background:**

The role of lung ultrasound (LUS) in evaluating the mid- and long-term prognoses of patients with COVID-19 pneumonia is not yet known. The objectives of this study were to evaluate associations between LUS signs at the time of screening and clinical outcomes 1 month after LUS and to assess LUS signs at the time of presentation with known risk factors for COVID-19 pneumonia.

**Methods:**

This was a retrospective study of data prospectively collected 1 month after LUS screening of 447 adult patients diagnosed with COVID-19 pneumonia. Sonographic examination was performed in screening tents with the participants seated. The LUS signs (B-lines > 2, coalescent B-lines, and subpleural consolidations) were captured in six areas of each hemithorax and a LUS aeration score was calculated; in addition, the categories of disease probability based on patterns of LUS findings (high-probability, intermediate-probability, alternate, and low-probability patterns) were evaluated. The LUS signs at patients’ initial evaluation were related to the following outcomes: symptomatology, the need for hospitalization or invasive mechanical ventilation (IMV), and COVID-19-related death.

**Results:**

According to the evaluations performed 1 month after LUS screening, 36 patients were hospitalised, eight of whom required intensive care unit (ICU) admission and three of whom died. The presence of coalescent B-lines was associated with the need for hospitalization (*p* = 0.008). The presence of subpleural consolidations was associated with dyspnoea (*p* < 0.0001), cough (*p* = 0.003), the need for hospitalization (*p* < 0.0001), the need for ICU admission (*p* < 0.0001), and death (*p* = 0.002). A higher aeration score was associated with dyspnoea (*p* < 0.0001), the need for hospitalization (*p* < 0.0001), the need for ICU admission (*p* < 0.0001), and death (*p* = 0.003). In addition, patients with a high-probability LUS pattern had a higher aeration score (*p* < 0.0001) and more dyspnoea (*p* = 0.024) and more often required hospitalization (*p* < 0.0001) and ICU admission (*p* = 0.031).

**Conclusions:**

In patients with COVID-19 pneumonia, LUS signs were related to respiratory symptoms 1 month after LUS screening. Strong relationships were identified between LUS signs and the need for hospitalization and death.

## Background

In the lungs, infection by severe acute respiratory syndrome coronavirus 2 (SARS-CoV-2) is characterized by severe pneumonia and/or acute respiratory distress syndrome (ARDS) in approximately 20% of patients, and mid- and long-term respiratory complications are beginning to be described [1]. However, data from previous coronavirus outbreaks, such as SARS and Middle East respiratory syndrome, suggest that some patients will have these complications after the acute phase of the disease [2]. The possible pulmonary sequelae include pulmonary vascular disease and interstitial lung disease; however, many other respiratory manifestations and changes in pulmonary function can be observed [3, 4].

Amid the coronavirus disease 2019 (COVID-19) pandemic and given the frequent lung involvement observed, new tools are needed to evaluate affected patients and to identify data with potential prognostic implications. Because lung ultrasound (LUS) is a fast test, it has been increasingly used as an alternative imaging method, and evidence supports its ability to identify lung lesions in COVID-19 [5–7]. In fact, LUS can be useful at various times, from screening for early diagnosis of lung involvement to decision-making about intensive care unit (ICU) treatment and respiratory support (e.g., mechanical ventilation, prone positioning, positive end-expiratory pressure, recruitment manoeuvres) [8–12]. Because of the strong association between LUS and computed tomography (CT) findings, LUS may be effective for diagnosing peripherally distributed lesions, which are characteristic of SARS-CoV-2 infection [13, 14]. In addition, LUS can identify the persistence of subclinical residual lung damage in COVID-19, even if patients meet the discharge criteria [15].

With the increasing number of patients who have survived COVID-19 worldwide, the most effective follow-up strategies for these patients must be determined. However, few studies have explored the follow-up of such patients after the acute phase of the disease. Severe disease is known to be associated with a higher probability of disability, and patients requiring ICU admission have been observed to have worse mid- and long-term outcomes, with physiological impairment and persistent radiological changes, than those not requiring ICU admission [3]. Because LUS has been increasingly used in the diagnosis of COVID-19 pneumonia, evidence of the prognostic role of LUS should be extended to the management of COVID-19 pneumonia. Thus, the objectives of this study were to evaluate associations between LUS signs at patients’ initial evaluation as a screening tool for pulmonary involvement due to COVID-19 and clinical outcomes 1 month after LUS and to assess LUS signs at the time of presentation with known risk factors for COVID-19 pneumonia.

## Materials and methods

### Participants

This was a retrospective study of data prospectively collected from LUS examinations performed on patients aged ≥ 18 years with fever and/or acute respiratory symptoms (dyspnoea, cough, and/or coryza) and diagnosed with COVID-19 confirmed by reverse-transcription polymerase chain reaction (RT-PCR). The patients were screened in tents installed in the courtyard of Piquet Carneiro Policlinic, State University of Rio de Janeiro, Rio de Janeiro, Brazil. Patients with LUS exams showing no pathological signs (absence of significant multiple and coalescent B-lines, absence of peripheral and large consolidations) were excluded. The results were analysed at the time of the initial LUS examination, and a clinical follow-up was conducted by phone 1 month after the initial LUS test. The outcomes studied included symptomatology, the need for hospitalization, the need for invasive mechanical ventilation (IMV), and COVID-19-related death. The study was approved by the National Research Ethics Committee of Brazil under number CAAE-30135320.0.0000.5259. All participants read the protocol and agreed to participate in the study.

### Lung ultrasound

**Fig. 1:**
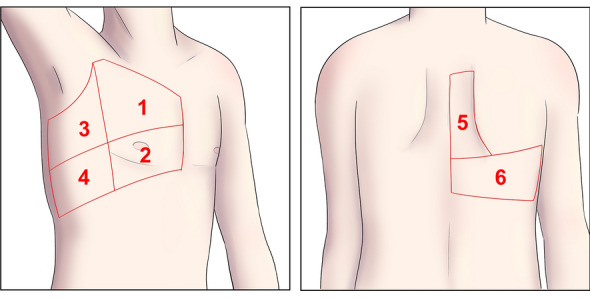
Representation of the 12 zones on the chest. **a** The anterior and axillary zones; **b** the posterior zones. The zones 1 and 2 are limited by the parasternal and anterior axillary lines. The zones 3 and 4 are limited by the anterior axillary and posterior axillary lines. Finally, the zones 5 and 6 are limited by the paravertebral and posterior axillary lines and by the contour of the scapula

We used an Aplio XG device (Toshiba Medical Systems, Tokyo, Japan) for the LUS examinations; the device was coupled to a 7.5–10-MHz multifrequency linear transducer or a 3.5- to 5-MHz convex transducer in B mode. The convex transducer was routinely used for analysis, while the linear transducer was used only when doubts remained regarding the analysis of the pleural surface. All LUS evaluations were carried out by a team of 6 pulmonologists employed at the policlinic with experience in the method (three with 13 years of experience, two with 11 years of experience, and one with 8 years of experience in ultrasound at the screening site). The LUS exams were performed with the participants in a sitting position since they were all outpatients and had no ventilatory or haemodynamic instability (individuals with respiratory symptoms were examined in screening tents). All LUS evaluations were performed by two examiners using a standard data collection form, and when disagreements occurred between the examiners, an agreement was reached through a collective discussion. Although no standard has been established with respect to specific scanning protocols, LUS signs were captured in six areas of each hemithorax following a 12-zone protocol [16, 17] as follows: two anterior, two lateral, and two posterior areas (Fig. 1). Each intercostal space of the upper and lower parts of the anterior, lateral, and posterior regions of the left and right chest walls were carefully examined. When evaluating pathological LUS signs, we searched for the following findings: B-lines > 2 (hyperechoic vertical artefacts arising from the pleural line, extending to the bottom of the screen without fading, and vacillating with lung movement, which occurs when the lung loses normal aeration but is not completely consolidated), coalescent B-lines (coalescence of many vertical artefacts to form more extended echogenic patterns corresponding to severe lung aeration loss), and subpleural consolidations (hypoechoic areas that appear as the subpleural density approaches the density of solid tissue, suggesting subpleural fluid-filled alveoli) [14, 18]. These abnormalities were evaluated separately and in combination to obtain an aeration score. To obtain the aeration score, we used a score for each area ranging from 0 to 3 according to the LUS sign as follows: B-lines > 2, 1 point; coalescent B-lines, 2 points; and consolidations, 3 points; the aeration score was calculated as the sum of these points and ranged between 0 and 36 [19]. In addition, we evaluated categories of disease probability based on patterns of LUS findings (high-probability, intermediate-probability, alternate, and low-probability patterns) [20–22].

### Statistical analysis

A nonparametric method was applied because the variables did not have a Gaussian distribution as indicated by rejection of the null hypothesis of a normal distribution by the Shapiro–Wilk test and graphical analysis of histograms. Thus, the most appropriate measures for summarizing the results were quartiles (the medians and interquartile ranges) for numerical data and the frequency (percentage) for categorical data. For numerical data, associations between pathological LUS signs, clinical findings detected 1 month after LUS, and poor outcomes were evaluated by the Mann–Whitney test for comparisons between two subgroups or Kruskal–Wallis ANOVA for comparisons between three subgroups (Dunn’s multiple comparison test was applied to identify which subgroups differed significantly from each other). For categorical data, associations were evaluated using the Chi-square or Fisher’s exact test. The significance level adopted was 5%. Statistical analysis was performed using the statistical software SAS 6.11 (SAS Institute, Inc., Cary, NC, USA).

## Results

Among the 460 patients eligible for inclusion in the study, 13 were lost to follow-up because they could not be reached by telephone. Among the 447 participants with COVID-19 pneumonia diagnosed by LUS included in the study, 305 (68.2%) were women, and the median age was 40 (34–50) years. The median time since symptom onset was 5 (2–7) days. The most prevalent comorbidities in this sample were hypertension (98, 21.9%) and diabetes (52, 11.6%). On LUS, the most frequent pathological sign was B-lines > 2, and the median aeration score at the time of COVID-19 pneumonia diagnosis was 4 (2–7). The demographic and clinical characteristics of the sample and the LUS data at the time of diagnosing pulmonary involvement are provided in Table 1.

**Table 1:** Sample characteristics at the time of diagnosis of pneumonia due to COVID-19 by LUS

Variables	Values
Demographic data	
Age (years)	40 (34–50)
Female sex	305 (68.2%)
BMI (kg/m^2^)	28.7 (35.5–32.7)
Comorbidities	
Hypertension	98 (21.9%)
Diabetes	52 (11.6)
Asthma	35 (7.8)
CHF	28 (6.3)
COPD	3 (0.67)
Others	110 (24.6)
Signs on lung ultrasound	
B-lines > 2	349 (78.1%)
Coalescent B-lines	228 (51%)
Subpleural consolidations	59 (13.2%)
Aeration score	4 (2–7)

Data are expressed as the median (interquartile range) or number (%)

*BMI* body mass index, *CHF* chronic heart failure, *COPD* chronic obstructive pulmonary disease

According to the evaluations performed 1 month after LUS screening for COVID-19 pneumonia, 36 (8.1%) patients required hospitalization due to pulmonary complications, with a median length of stay of 7 (4–10) days; among those who required hospitalization, 8 (1.8%) required admission to the ICU, and 3 (0.7%) died. In the sample studied 1 month after the initial evaluation, 121 (27.1%) reported general fatigue, while 62 (13.9%) complained of dyspnoea. Cough and fever were reported by 39 (8.7%) and 3 (0.7%) patients, respectively.

**Fig. 2:**
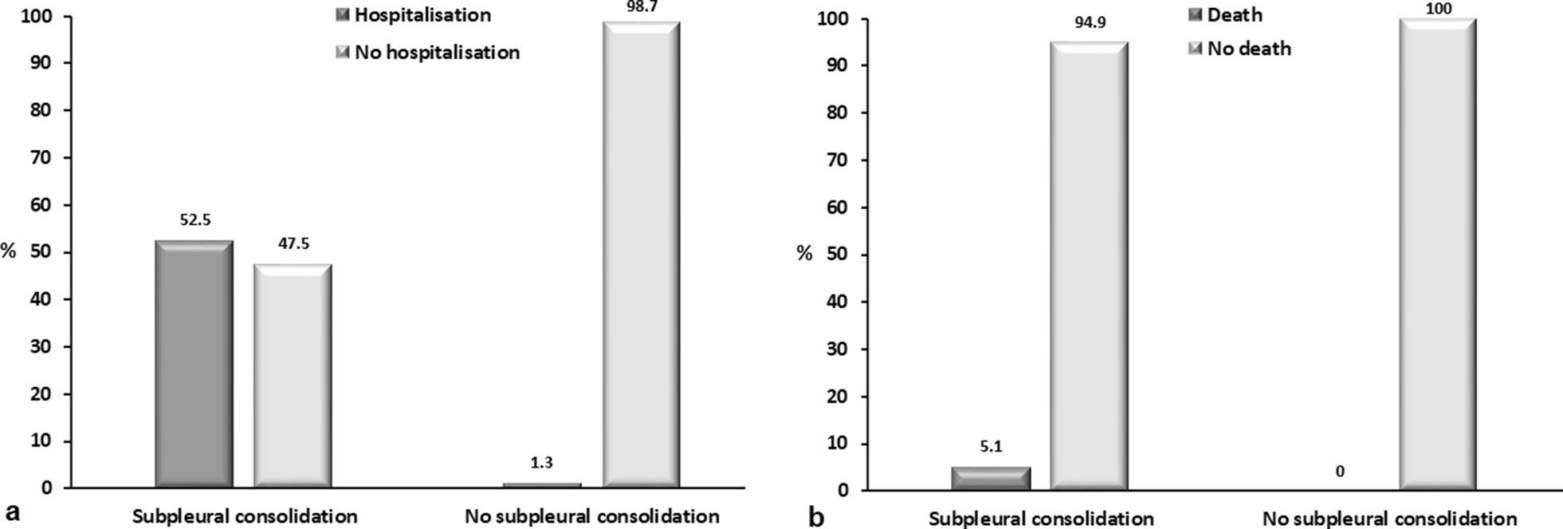
Relationships between the presence of subpleural consolidation on lung ultrasound and the outcomes of **a** hospitalization (*p* < 0.0001) and **b** death (*p* = 0.002)

We evaluated associations between LUS findings at the time of screening, clinical outcomes, and the presence of symptoms 1 month after LUS. In this analysis, the presence of B-lines > 2 was not associated with any clinical finding or outcome. However, the presence of coalescent B-lines was associated with older age (*p* = 0.035), a higher body mass index (*p* = 0.027), diabetes (*p* = 0.027), dyspnoea (*p* = 0.037), and the need for hospitalization (*p* = 0.008). The presence of subpleural consolidations on LUS was associated with diabetes (*p* = 0.002), chronic heart failure (CHF, *p* = 0.021), general fatigue (*p* = 0.013), dyspnoea (*p* < 0.0001), cough (*p* = 0.003), fever (*p* = 0.043), the need for hospitalization (*p* < 0.0001), the need for ICU admission (*p* < 0.0001), and death (*p* = 0.002) (Fig. 2).

**Fig. 3:**
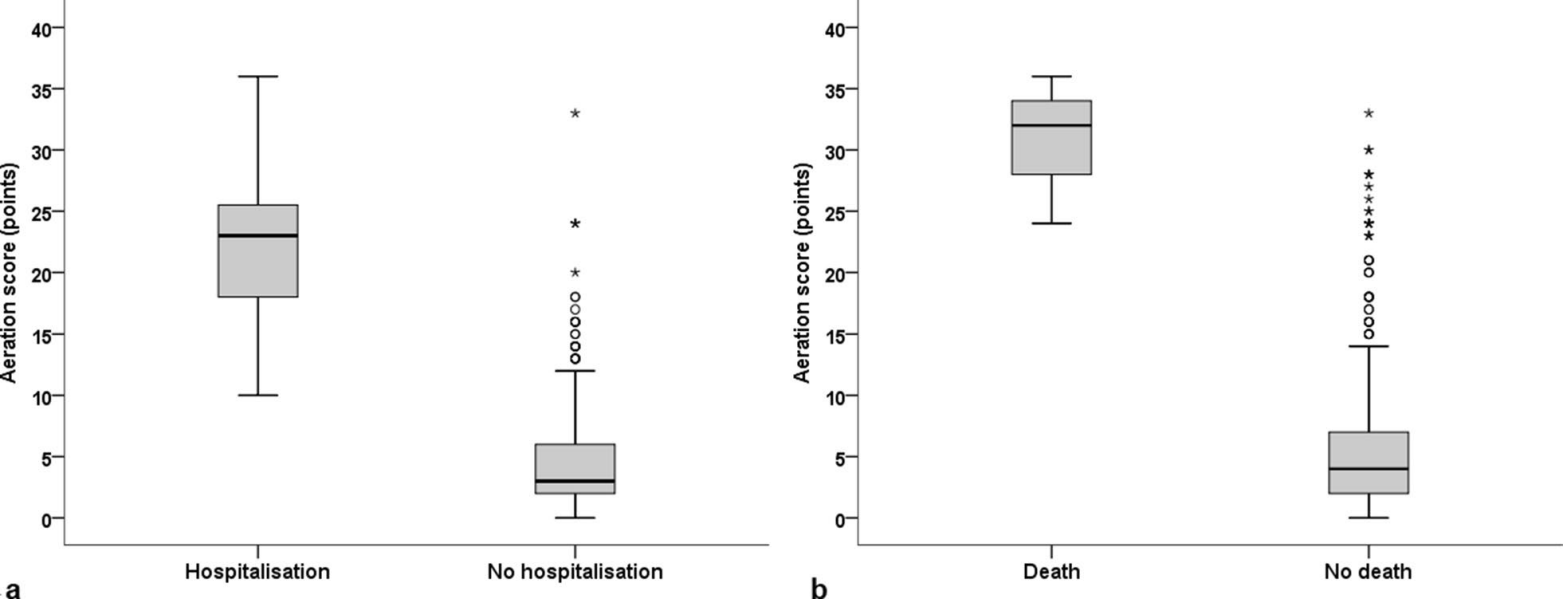
Relationships between the aeration score on lung ultrasound and the outcomes of **a** hospitalization (*p* < 0.0001) and **b** death (*p* = 0.003)

We evaluated the relationships of the LUS aeration score with clinical outcomes and symptoms 1 month after ultrasound examination. In this analysis, a higher aeration score was associated with hypertension (*p* = 0.032), diabetes (*p* = 0.0001), dyspnoea (*p* < 0.0001), cough (*p* = 0.041), the need for hospitalization (*p* < 0.0001), the need for ICU admission (*p* < 0.0001), and death (*p* = 0.003) (Fig. 3).

Additionally, we evaluated categories of the probability of COVID-19 based on patterns of LUS signals. In this analysis, 234 cases (52.3%) had high-probability patterns, 180 cases (40.3%) had intermediate-probability patterns, and 33 cases (7.4%) had alternate LUS patterns; no participant had low-probability patterns. Although all participants were positive for COVID-19 by RT-PCR, the alternate LUS patterns were as follows: chronic heart failure (*n* = 18), bacterial pneumonia (*n* = 6), tuberculosis sequelae (*n* = 4), systemic sclerosis (*n* = 3), and rheumatoid arthritis (*n* = 2). Interestingly, we observed that patients with a high-probability LUS pattern had a higher aeration score (*p* < 0.0001) and more dyspnoea (*p* = 0.024) and more often required hospitalization (*p* < 0.0001) and ICU admission (*p* = 0.031) (Table 2).

**Table 2:** Comparisons between disease probability categories based on LUS signal patterns

Variables	High-probability pattern (*n* = 234)	Intermediate-probability pattern (*n* = 180)	Alternate pattern (*n* = 33)	*p*-value
LUS aeration score	7 (4–13.3)^a,b^	2 (1–2)^b^	3 (1–4)	** < 0.0001**
Symptoms 1 month after LUS				
General fatigue	61 (26.1)	48 (26.7)	12 (36.4)	0.47
Dyspnoea	39 (16.7)^a^	16 (8.9)	7 (21.2)	**0.024**
Cough	21 (8.9)	9 (5)	9 (27.3)	0.29
Fever	2 (0.9)	1 (0.6)	0 (0)	0.99
Poor outcomes				
Need for hospitalization	36 (15.4)^a,b^	0 (0)	0 (0)	** < 0.0001**
Need for ICU admission	8 (3.4)^a^	0 (0)	0 (0)	**0.031**
Death	3 (1.3)	0 (0)	0 (0)	0.41

Data are expressed as the number (%) except for the aeration score, which is expressed as the median (interquartile range)

Bold values indicate significant differences

*ICU* intensive care unit

^a^Significantly different from the intermediate-probability pattern

^b^Significantly different from the alternate pattern

## Discussion

LUS has played a critical role in the pandemic caused by SARS-CoV-2, with growing evidence of its usefulness both to screen for lung involvement and to evaluate disease severity. Sequential LUS has also been used as an efficient tool to monitor the progression of lung lesions in individuals with more severe lung disease. Despite all these indications, the prognostic power of LUS in COVID-19 is also important to evaluate. Here, we searched for correlations between pathological LUS signs and patient outcomes. The main results of the present study were that 1 month after LUS screening, LUS signs (especially subpleural consolidations) were associated with the presence of persistent respiratory symptoms and general fatigue. The LUS findings strongly predicted the need for hospitalization (including in the ICU) and death. In addition, the LUS aeration score was correlated with the persistence of respiratory manifestations at 1 month after ultrasound examination. Finally, we also observed a strong relationship between aeration scores and poor outcomes, such as the need for hospitalization (including in the ICU) and death. To our knowledge, this study is the first to evaluate the relationship between pathological LUS signs and 1-month clinical outcomes.

The mid- and long-term complications of COVID-19, including those related to the respiratory system, should be identified, and affected patients require follow-ups by appropriate services. In the present study, we observed that respiratory manifestations (such as dyspnoea and cough) and systemic manifestations (such as fever and general fatigue) were associated with LUS signs diagnosed 1 month before the prospective evaluation. Since ultrasound equipment is increasingly available and LUS can be performed at the bedside within a few minutes and in patients with mild disease or even in unstable patients [23], we believe that these interrelationships may be of great clinical interest in the context of the COVID-19 pandemic. Although LUS signs may not be specific for COVID-19 compared to some other lung diseases, the identification of certain patterns in the epidemiological context of the pandemic can certainly help clinicians to identify individuals likely to exhibit worsening clinical conditions and to develop sequelae after resolution of the acute phase of the disease [24, 25].

The clinical significance of B-lines depends mainly on their quantity (the number of B-lines per area examined and the presence or absence of confluent B-lines) and is usually associated with interstitial syndromes [2]. As the disease progresses, the air content decreases and lung density and the number of B-lines increase, leading to confluent areas equivalent to ground-glass opacities (GGOs) on CT [2, 24]. In the present study, we observed that coalescent B-lines were associated with older age, obesity, and diabetes, which are considered risk factors for COVID-19 exacerbation [4, 26, 27]. However, the strongest associations with persistent respiratory symptoms 1 month after LUS were observed for previously diagnosed subpleural consolidations. In fact, as the pneumonic process progresses in COVID-19, lung density increases even more because of alveolar infiltration with inflammatory cells, causing loss of aeration and consolidation areas [2, 24]. Thus, the finding of subpleural consolidations on LUS may not only predict worsening of symptoms in the course of the disease [23] but may also indicate a more severe course in terms of pulmonary sequelae in SARS-CoV-2 infection.

The use of a scoring system to assess pathological LUS signs in patients infected with SARS-CoV-2 has been increasingly widespread during the pandemic [11, 28]. In our study, we observed that the LUS aeration score was strongly related to dyspnoea and cough 1 month after ultrasound screening. Interestingly, Lichter et al. [29] evaluated 120 consecutive patients with COVID-19 who underwent LUS within 24 h after admission. These authors observed that clinical deterioration was associated with increased follow-up LUS scores, mainly due to loss of aeration in anterior lung segments. Using an LUS scoring system and the need for supplemental oxygen at the time of examination, Manivel et al. [30] proposed a protocol to assist clinicians with decision-making in patients with COVID-19 and to facilitate provision planning within emergency departments. Because our study examined 1-month outcomes, we think that our results provide additional evidence that the LUS score can be used as a prediction tool for clinical outcomes of the disease after the acute phase and can be used as an additional tool for clinical reasoning.

In COVID-19, lung lesions play a key role in determining the clinical course and prognosis [31]. In this sense, one of the objectives of the current study was to evaluate the correlations of LUS findings with poor outcomes. We observed that the pathological LUS signs (particularly subpleural consolidations) and a higher aeration score were associated with the need for hospitalization, the need for ICU admission, and death. In line with our findings, some researchers have observed that early LUS signs (including the LUS score) are effective for assessing the need for prolonged hospitalization [15, 32]. Other studies have shown the potential of LUS to stratify early risk in COVID-19 patients who visit emergency departments according to mortality risk and the need for IMV, with this risk being higher in individuals with more pathological lung areas [29, 33, 34]. Given the scarcity of equipment and trained health professionals, the use of a relatively simple diagnostic procedure that does not use ionizing radiation, such as LUS, may have clinical and public health implications [34]. In contrast to studies evaluating short-term consequences, our study assessed 1-month outcomes, which may have important implications from the perspective of utilizing scarce resources in disadvantaged socioeconomic areas.

When using LUS, the criteria for positivity must be defined considering the possibility of alternative diagnoses and levels of probability [20, 21]. Because LUS signs are nonspecific, LUS cannot be used alone to establish a definitive diagnosis of COVID-19 infection [35]. In fact, almost 10% of our cases had LUS patterns more consistent with other diagnoses (including cardiogenic pulmonary oedema and bacterial pneumonia), although the diagnosis of COVID-19 was confirmed in all participants by RT-PCR. Importantly, our study showed that patients with high-probability LUS patterns had higher aeration scores and more dyspnoea 1 month after LUS screening and more often required hospitalization and ICU admission. Although more studies are needed, the use of categories of the probability of COVID-19 pneumonia can be an interesting strategy to predict the evolution of the disease in the short and medium term.

Several limitations in our study should be noted. First, our study was conducted at a single reference centre during the screening of COVID-19 pneumonia; therefore, generalization of our results to other periods of the pandemic should be executed with caution. However, with the emergence of the second wave of the pandemic in many regions of the world, the role of LUS may become even more prominent in the process of implementing local and global measures efficiently. Second, ultrasound evaluates only approximately 1/16 of the total lung and detects only changes closely related to the pleural surface [36]; therefore, other chest imaging methods may have a greater prognostic role than LUS. Third, we performed only a single evaluation by LUS; serial LUS examinations can be useful for tracking the clinical trajectory of an apparently unpredictable disease course, thus improving the prediction of clinical outcomes.

## Conclusion

In patients with COVID-19 pneumonia, LUS signs, including the aeration score, were related to respiratory and systemic symptoms 1 month after ultrasound screening examination. In these patients, a strong relationship was also found between LUS findings and poor outcomes, such as the need for hospitalization (including in the ICU) and death. Future studies should evaluate the relationship between LUS signs at the time of diagnosing lung involvement and long-term consequences.

## Data Availability

All data generated or analysed during this study are included in this published article.

## Abbreviations

SARS-CoV-2: Severe acute respiratory syndrome coronavirus 2
ARDS: Acute respiratory distress syndrome
COVID-19: Coronavirus disease 2019
LUS: Lung ultrasound
ICU: Intensive care unit
CT: Computed tomography
RT-PCR: Reverse-transcription polymerase chain reaction
IMV: Invasive mechanical ventilation
GGO: Ground-glass opacities
